# Di­chlorido­{(*E*)-*N*,*N*-dimethyl-2-[phen­yl(pyridin-2-yl)methyl­idene]hydrazine-1-carbo­thio­amide}cadmium(II)

**DOI:** 10.1107/S241431462500464X

**Published:** 2025-05-30

**Authors:** Christian S. Parry, Bernard Kwabi-Addo, Timothy R. Ramadhar, Raymond J. Butcher

**Affiliations:** a Department of Microbiology College of Medicine Howard University, Washington, DC 20059 USA; b Department of Biochemistry College of Medicine Howard University, Washington, DC 20059 USA; c Department of Chemistry College of Arts and Science Howard University, Washington, DC 20059 USA; Goethe-Universität Frankfurt, Germany

**Keywords:** crystal structure, post-industrial pollution, riverways and food chain, thio­semicarbazone heterocycle ligands, heavy metals

## Abstract

The structure of the title compound, [CdCl_2_(C_15_H_16_N_4_S)], at 100 K has monoclinic (*P*2_1_/*c*) symmetry. The compound has a layer structure and is a 1:1 complex of the organic ligand and cadmium chloride. The ligand, 3,3-dimethyl-1-[(*E*)-[phen­yl(pyridine-2-yl)methyl­idene]amino]­thio­urea (*L*, Bp44mT), is of inter­est with respect to anti­cancer activity, anti­viral properties and potential use in conditions of iron overload, from hemochromatosis or from multiple transfusions in hematological disorders such as sickle cell disease or beta thalassemia. This study is aimed at uncovering the basis of selectivity of the ligand as a chelator and for lead optimization. We also examine the ligand’s potential use in treating heavy metal poisoning from cadmium, arsenic, lead or mercury, and for environmental remediation. The crystal structure exhibits no inter­molecular or intra­molecular hydrogen bonding with the supra­molecular features being driven by hydro­phobic, π–π and long-range dispersion forces.

## Abstract 2

The structure of the cadmium-bound model ligand, 3,3-dimethyl-1-[(*E*)-[phen­yl(pyridine-2-yl)methyl­idene]amino]­thio­urea (Bp44mT), which is used across medicinal chemistry and in metal-based nanoparticles research has been determined and analyzed. This complex is used to gain insight to the specificity and selectivity of the ligand and to model how 3,3-dimethyl-1-[(*E*)-[phen­yl(pyridine-2-yl)methyl­idene]amino]­thio­urea might be used in chelation to counter metal poisoning and for environmental remediation.

## Graphical abstract

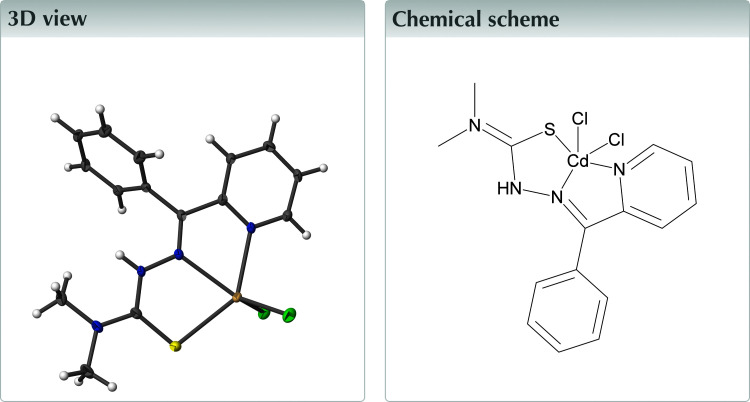

## Structure description

**Figure 1:**
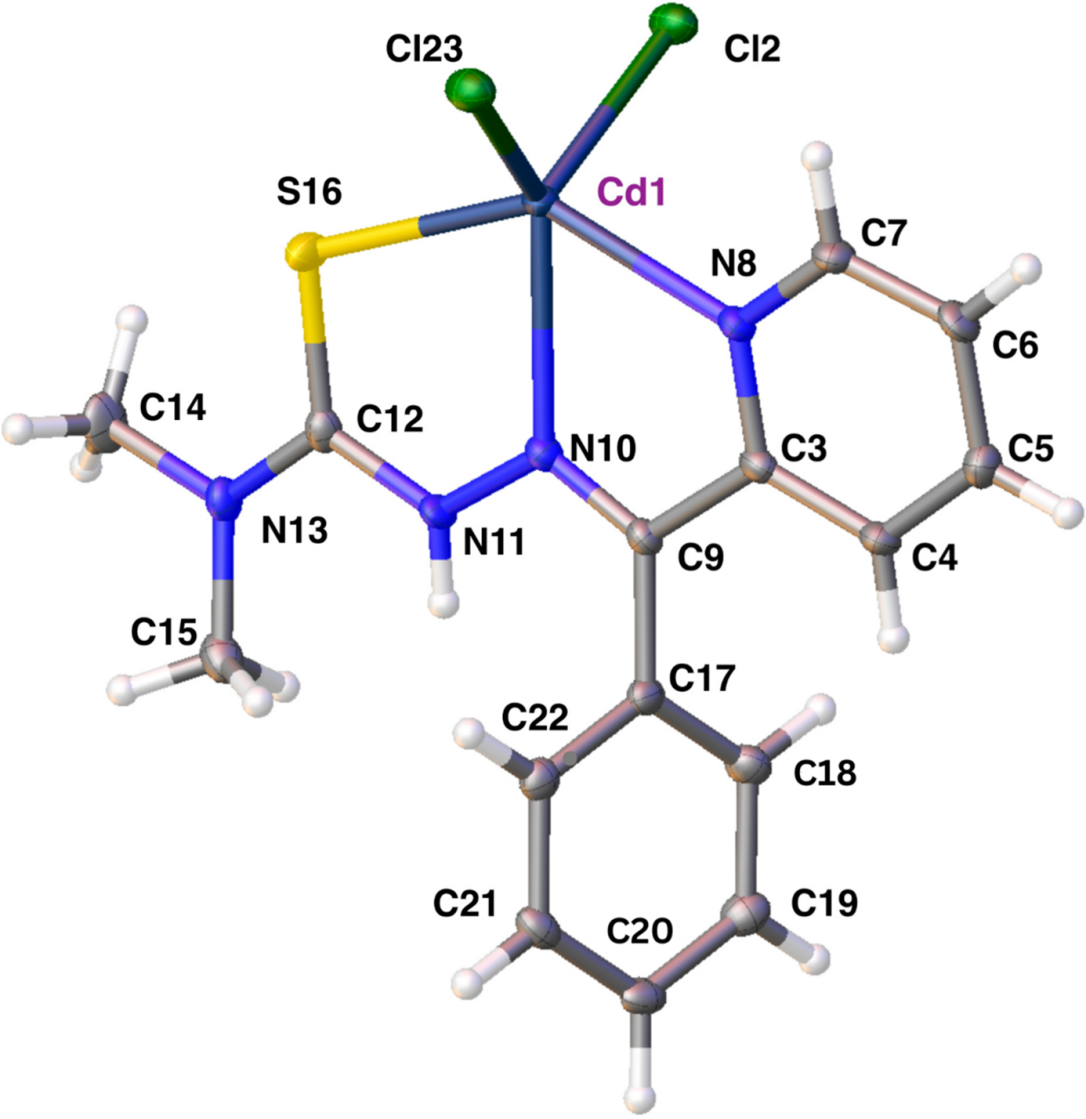
Labeled structure of the title compound. Atomic displacement parameters are set at the 30% probability level.

Waste and corrosion products from shuttered industrial plants are contaminating food and waterways in once bustling communities. The major culprits are lead and cadmium, but also mercury and arsenic. The accumulation of heavy metals in sufficient concentration is toxic, causing metal poisoning with serious damage to organs and tissues. Children are particularly vulnerable. Worse, metal accumulation is passed on through the soil, food, fish and other aqua­tic organisms with environmental, public health and economic consequences. The ligand 3,3-dimethyl-1-[(*E*)-[phen­yl(pyridine-2-yl)methyl­idene]amino]­thio­urea (*L*), a model iron chelator, commonly known as 2-benzoyl­pyridine-4,4-dimethyl-3-thio­semicarbazone (Bp44mT) (Yu *et al.*, 2012 ▸), has been used to form a cadmium-bound complex. The ligand binds to cadmium in a 1:1 ligand:metal ratio. Cadmium binding is through the ligand tridentate donor atoms N8, N10 and S16 with the metal being further coordinated by the two Cl^−^ anions from the salt. Two planes define the structure of the compound: the coordinate bonds formed between cadmium and the ligand (N, N′ and S) constrain the compound, except the phenyl ring, to the plane of the pyridine ring; the phenyl ring forms the other plane. This structure is congruent with our prior structure of the unbound ligand in which the hydrogen bond between hydrazine N and pyridine N′ similarly enforces planarity (Parry *et al.*, 2025 ▸). R.m.s.d. values for atoms that define the plane of the pyridine ring are 0.007 Å and 0.004 Å for those that define the phenyl ring. The distance between their centroids is 4.7572 (19) Å; the angle of the phenyl ring normal to the pyridine plane normal is 67.27 (11)°. Selected bond lengths and angles are given in Table 1 ▸ and the molecular structure is shown in Fig. 1 ▸..

The title structure is sharply distinct from a 2:1 ligand: cadmium structure of bis­{*N*,*N*-dimethyl-*N′*-[phen­yl(pyridin-2-yl)methyl­idene]carbamohydrazono­thio­ato}cadmium(II) [refcode BIHTAQ (Fang *et al.*, 2018 ▸) in the Cambridge Structural Database (Groom *et al.*, 2016 ▸)]. In BIHTAQ, cadmium(II) binds to the ligand, *N*,*N*-dimethyl-2-[phen­yl(pyridin-2-yl)methyl­idene]hydrazine-1-carbo­thio­amide, *via* the tridentate donors N, N′ and S in a six-coordinate (octa­hedral) mode. Cadmium coordination in the present structure is five-coordinate with a coordination mode in between a distorted square-pyramidal and a distorted trigonal–bipyramidal structure.

**Figure 2:**
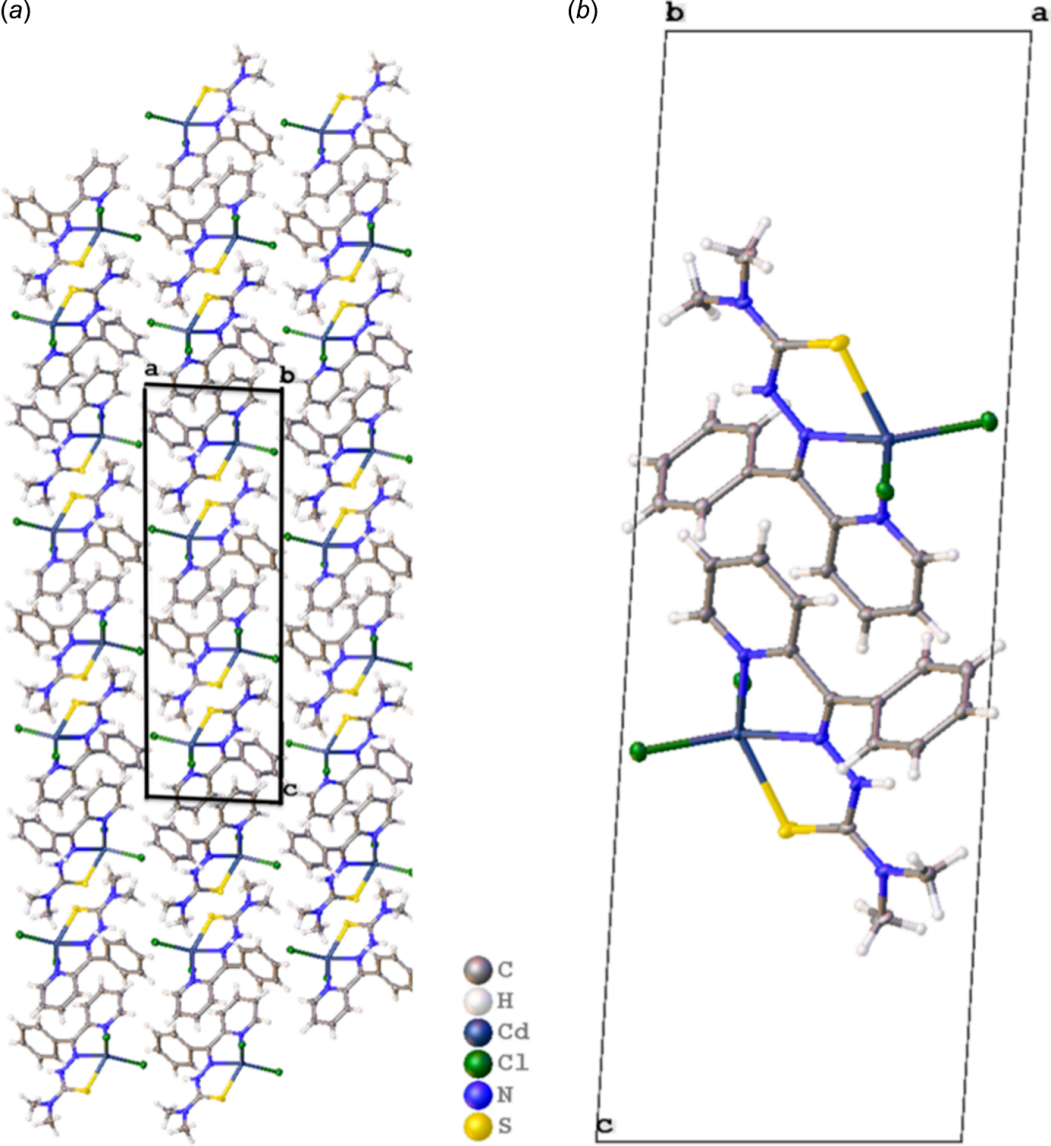
Packing scheme. (*a*) The packing is dominated by hydro­phobic inter­actions. The unit cell is shown. (*b*) The basic inter­action is shown in isolation within the unit cell.

**Figure 3:**
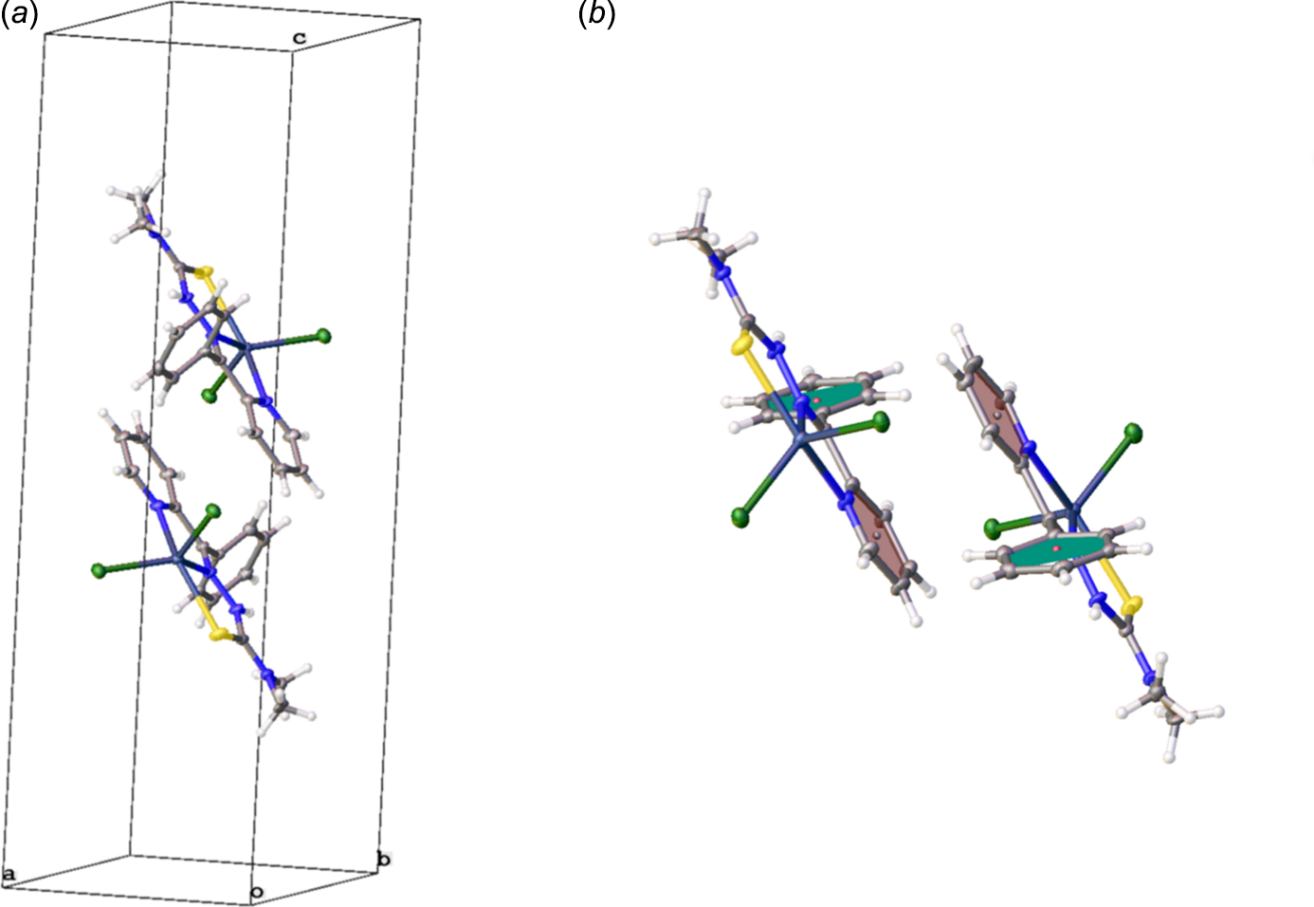
The π–π inter­action component of the packing. (*a*) The basic π–π inter­action is shown within the unit cell. (*b*) Another view of the π–π inter­action.

There are no hydrogen bonds in the structure of the compound. The forces effectuating supra­molecular features are hydro­phobicity, π–π inter­actions [*Cg*⋯*Cg*(1 − *x*, 1 − *y*, 1 − *z*) = 3.9912 (15) Å, where *Cg* is the centroid of the N8/C3–C7 ring] and long-range dispersion effects. Fig. 2 ▸*a* shows the aliphatic stem of the ligand packing against the carbon groups of the heterocyclic groups and to hydro­phobic sulfur atoms (Chibowski & Hołysz, 1989 ▸) while Fig. 2 ▸*b* offers a more detailed view. We searched and identified the structural unit propagating π–π bonding. This is isolated and shown within the unit cell (Fig. 3 ▸*a*). It involves two complexes lying in adjacent planes. Their aromatic rings are slightly offset – pyridine group to pyridine group and phenyl ring to phenyl ring – but are able to contribute significant favorable inter­actions to the packing. A different view is shown in Fig. 3 ▸*b*, in which the planes defining pyridine are shown in chocolate brown and the plane defining the phenyl ring is colored green. Respective centroids are marked.

## Synthesis and crystallization

Ligand *L* was synthesized for us by custom order by Enamine (Monmouth Junction, NJ) and delivered > 95% pure (Parry *et al.*, 2025 ▸). We obtained crystals by vapor diffusion using aceto­nitrile as solvent and ether as precipitant.

## Refinement

Crystal data, data collection and structure refinement details are summarized in Table 2 ▸.

## Supplementary Material

Crystal structure: contains datablock(s) I. DOI: 10.1107/S241431462500464X/bt4171sup1.cif

Structure factors: contains datablock(s) I. DOI: 10.1107/S241431462500464X/bt4171Isup2.hkl

CCDC reference: 2427465

Additional supporting information:  crystallographic information; 3D view; checkCIF report

## Figures and Tables

**Table 1:** Selected geometric parameters (Å, °)

Cd1—Cl2	2.4430 (6)	Cd1—N8	2.352 (2)
Cd1—S16	2.6001 (6)	Cd1—N10	2.403 (2)
Cd1—Cl23	2.4832 (6)	S16—C12	1.700 (3)
			
Cl2—Cd1—S16	104.61 (2)	N10—Cd1—Cl2	120.90 (5)
Cl2—Cd1—Cl23	109.64 (2)	N10—Cd1—S16	73.20 (5)
Cl23—Cd1—S16	108.43 (2)	N10—Cd1—Cl23	127.43 (5)
N8—Cd1—Cl2	99.39 (5)	C12—S16—Cd1	102.86 (9)
N8—Cd1—S16	140.28 (5)	C3—N8—Cd1	119.79 (16)
N8—Cd1—Cl23	92.25 (5)	C7—N8—Cd1	121.53 (17)
N8—Cd1—N10	67.36 (7)	C7—N8—C3	118.5 (2)

**Table 2:** Experimental details

Crystal data	
Chemical formula	[CdCl_2_(C_15_H_16_N_4_S)]
*M*_r_	467.68
Crystal system, space group	Monoclinic, *P*2_1_/*c*
Temperature (K)	100
*a*, *b*, *c* (Å)	8.58301 (8), 7.87355 (7), 26.1616 (2)
β (°)	93.6036 (8)
*V* (Å^3^)	1764.47 (3)
*Z*	4
Radiation type	Cu *K*α
μ (mm^−1^)	13.83
Crystal size (mm)	0.6 × 0.2 × 0.2

Data collection	
Diffractometer	XtaLAB Synergy, Dualflex, HyPix-6000
Absorption correction	Multi-scan (*CrysAlis PRO*; Rigaku OD, 2023 ▸)
*T*_min_, *T*_max_	0.489, 1.000
No. of measured, independent and observed [*I* > 2σ(*I*)] reflections	11604, 3603, 3407
*R*_int_	0.034
(sin θ/λ)_max_ (Å^−1^)	0.634

Refinement	
*R*[*F*^2^ > 2σ(*F*^2^)], *wR*(*F*^2^), *S*	0.027, 0.071, 1.05
No. of reflections	3603
No. of parameters	210
H-atom treatment	H-atom parameters constrained
Δρ_max_, Δρ_min_ (e Å^−3^)	0.62, −0.86

Computer programs: *CrysAlis PRO* (Rigaku OD, 2023 ▸), *SUPERFLIP* (Palatinus & Chapuis, 2007 ▸; Palatinus & van der Lee, 2008 ▸; Palatinus *et al.*, 2012 ▸), *SHELXL2018/3* (Sheldrick, 2015 ▸) and *OLEX2* (Dolomanov *et al.*, 2009 ▸; Bourhis *et al.*, 2015 ▸).

## full crystallographic data

### Dichlorido{(E)-N,N-dimethyl-2-[phenyl(pyridin-2-yl)methylidene]hydrazine-1-carbothioamide}cadmium(II) Crystal data

**Table:**

[CdCl_2_(C_15_H_16_N_4_S)]	*F*(000) = 928
*M**_r_* = 467.68	*D*_x_ = 1.761 Mg m^−^^3^
Monoclinic, *P*2_1_/*c*	Cu *K*α radiation, λ = 1.54184 Å
*a* = 8.58301 (8) Å	Cell parameters from 7680 reflections
*b* = 7.87355 (7) Å	θ = 3.4–76.5°
*c* = 26.1616 (2) Å	µ = 13.83 mm^−^^1^
β = 93.6036 (8)°	*T* = 100 K
*V* = 1764.47 (3) Å^3^	Irregular, metallic yellowish brown
*Z* = 4	0.6 × 0.2 × 0.2 mm

### Dichlorido{(E)-N,N-dimethyl-2-[phenyl(pyridin-2-yl)methylidene]hydrazine-1-carbothioamide}cadmium(II) Data collection

**Table:**

XtaLAB Synergy, Dualflex, HyPix-6000 diffractometer	3603 independent reflections
Radiation source: micro-focus sealed X-ray tube, PhotonJet (Cu) X-ray Source	3407 reflections with *I* > 2σ(*I*)
Mirror monochromator	*R*_int_ = 0.034
Detector resolution: 10.0000 pixels mm^-1^	θ_max_ = 77.8°, θ_min_ = 3.4°
ω scans	*h* = −10→10
Absorption correction: multi-scan (CrysAlisPro; Rigaku OD, 2023)	*k* = −9→5
*T*_min_ = 0.489, *T*_max_ = 1.000	*l* = −33→29
11604 measured reflections	

### Dichlorido{(E)-N,N-dimethyl-2-[phenyl(pyridin-2-yl)methylidene]hydrazine-1-carbothioamide}cadmium(II) Refinement

**Table:**

Refinement on *F*^2^	Primary atom site location: iterative
Least-squares matrix: full	Hydrogen site location: inferred from neighbouring sites
*R*[*F*^2^ > 2σ(*F*^2^)] = 0.027	H-atom parameters constrained
*wR*(*F*^2^) = 0.071	*w* = 1/[σ^2^(*F*_o_^2^) + (0.0404*P*)^2^ + 0.8003*P*] where *P* = (*F*_o_^2^ + 2*F*_c_^2^)/3
*S* = 1.05	(Δ/σ)_max_ = 0.001
3603 reflections	Δρ_max_ = 0.62 e Å^−^^3^
210 parameters	Δρ_min_ = −0.86 e Å^−^^3^
0 restraints	

### Dichlorido{(E)-N,N-dimethyl-2-[phenyl(pyridin-2-yl)methylidene]hydrazine-1-carbothioamide}cadmium(II) Special details

**Table:**

Experimental. Crystals were harvested, mounted on the goniometer and centered. Diffraction data was collected from a Rigaku Oxford Synergy S Dual Source Diffractometer (Rigaku USA, The Woodlands, TX) driven by *CrysAlis PRO* (Oxford Diffraction, Yarnton UK). The crystal was kept at 100 K in a stream of liquid nitrogen (Oxford Cryosystems, Oxford, UK). Diffraction intensity was collected on a HyPix-6000 Detector (Rigaku USA, The Woodlands, TX). Collected X-ray diffraction data was integrated and finalized in *CrysAlis PRO*. The structure was solved using the *SUPERFLIP* charge flipping algorithm (Palatinus & Chapuis, 2007; Palatinus, & van der Lee, 2008; Palatinus *et al.*, 2012) implemented in *OLEX2*.solve 1.5 (Bourhis *et al.*, 2015) and refined with *SHELXL* (Sheldrick, 2015) running under *OLEX2*–1.5 (Dolomanov *et al.*, 2009).
Geometry. All esds (except the esd in the dihedral angle between two l.s. planes) are estimated using the full covariance matrix. The cell esds are taken into account individually in the estimation of esds in distances, angles and torsion angles; correlations between esds in cell parameters are only used when they are defined by crystal symmetry. An approximate (isotropic) treatment of cell esds is used for estimating esds involving l.s. planes.
Refinement. No restraints were used. Non-hydrogen atoms were refined with anisotropic thermal parameters, and hydrogen atoms were placed and allowed to refine using a riding model. Methyl groups were idealized and refined as rotating groups: C14(H14A, H14B, H14C) and C15(H15A, H15B, H15C).

### Dichlorido{(E)-N,N-dimethyl-2-[phenyl(pyridin-2-yl)methylidene]hydrazine-1-carbothioamide}cadmium(II) Fractional atomic coordinates and isotropic or equivalent isotropic displacement parameters (Å^2^)

**Table:**

	*x*	*y*	*z*	*U*_iso_*/*U*_eq_	
Cd1	0.32068 (2)	0.37636 (2)	0.63193 (2)	0.01458 (8)	
Cl2	0.32122 (7)	0.10628 (8)	0.58597 (2)	0.02127 (14)	
S16	0.47203 (8)	0.31437 (9)	0.71916 (2)	0.02291 (15)	
Cl23	0.04850 (7)	0.45555 (8)	0.64866 (2)	0.02132 (14)	
N8	0.3247 (2)	0.5639 (3)	0.56203 (8)	0.0157 (4)	
C21	0.8095 (3)	1.0446 (3)	0.64425 (10)	0.0190 (5)	
H21	0.806392	1.133222	0.668803	0.023*	
N13	0.7411 (2)	0.4475 (3)	0.75560 (8)	0.0170 (4)	
N10	0.5467 (2)	0.5572 (3)	0.63645 (7)	0.0154 (4)	
C14	0.7436 (3)	0.3269 (4)	0.79814 (10)	0.0246 (6)	
H14A	0.695338	0.378944	0.827270	0.037*	
H14B	0.851899	0.296266	0.808253	0.037*	
H14C	0.685370	0.224531	0.787421	0.037*	
C17	0.6955 (3)	0.7942 (3)	0.60394 (9)	0.0157 (5)	
C22	0.6928 (3)	0.9225 (3)	0.64090 (9)	0.0174 (5)	
H22	0.610953	0.925997	0.663700	0.021*	
C9	0.5675 (3)	0.6670 (3)	0.60118 (9)	0.0146 (5)	
C18	0.8178 (3)	0.7886 (3)	0.57137 (10)	0.0197 (5)	
H18	0.820495	0.701472	0.546315	0.024*	
C20	0.9317 (3)	1.0370 (3)	0.61143 (10)	0.0201 (5)	
H20	1.012337	1.119755	0.613958	0.024*	
N11	0.6548 (2)	0.5470 (3)	0.67711 (7)	0.0163 (4)	
H11	0.739897	0.609484	0.677388	0.020*	
C19	0.9353 (3)	0.9090 (4)	0.57527 (10)	0.0206 (5)	
H19	1.018646	0.903866	0.553075	0.025*	
C12	0.6319 (3)	0.4411 (3)	0.71716 (9)	0.0158 (5)	
C6	0.2184 (3)	0.6590 (4)	0.48022 (10)	0.0196 (5)	
H6	0.136360	0.654286	0.454139	0.023*	
C3	0.4487 (3)	0.6669 (3)	0.55744 (9)	0.0145 (5)	
C4	0.4631 (3)	0.7673 (3)	0.51456 (9)	0.0167 (5)	
H4	0.551360	0.839065	0.512169	0.020*	
C5	0.3471 (3)	0.7620 (3)	0.47506 (9)	0.0189 (5)	
H5	0.356063	0.827975	0.444983	0.023*	
C15	0.8625 (3)	0.5789 (4)	0.75933 (10)	0.0205 (5)	
H15A	0.896630	0.596802	0.795375	0.031*	
H15B	0.820319	0.685232	0.744716	0.031*	
H15C	0.951580	0.542510	0.740372	0.031*	
C7	0.2124 (3)	0.5627 (4)	0.52455 (10)	0.0196 (5)	
H7	0.123750	0.492540	0.528295	0.023*	

### Dichlorido{(E)-N,N-dimethyl-2-[phenyl(pyridin-2-yl)methylidene]hydrazine-1-carbothioamide}cadmium(II) Atomic displacement parameters (Å^2^)

**Table:**

	*U*^11^	*U*^22^	*U*^33^	*U*^12^	*U*^13^	*U*^23^
Cd1	0.01462 (11)	0.01564 (12)	0.01332 (11)	−0.00527 (6)	−0.00038 (7)	0.00023 (6)
Cl2	0.0209 (3)	0.0201 (3)	0.0232 (3)	−0.0047 (2)	0.0051 (2)	−0.0055 (2)
S16	0.0256 (3)	0.0260 (3)	0.0165 (3)	−0.0107 (3)	−0.0039 (2)	0.0071 (2)
Cl23	0.0186 (3)	0.0212 (3)	0.0244 (3)	−0.0023 (2)	0.0033 (2)	−0.0040 (2)
N8	0.0152 (10)	0.0182 (10)	0.0131 (9)	−0.0042 (8)	−0.0033 (7)	0.0001 (8)
C21	0.0206 (12)	0.0180 (13)	0.0177 (11)	−0.0035 (10)	−0.0052 (9)	−0.0031 (10)
N13	0.0197 (10)	0.0188 (11)	0.0121 (9)	0.0014 (8)	−0.0027 (7)	0.0012 (8)
N10	0.0146 (9)	0.0180 (11)	0.0132 (9)	−0.0030 (8)	−0.0028 (7)	0.0011 (8)
C14	0.0309 (14)	0.0243 (14)	0.0174 (12)	0.0042 (12)	−0.0079 (10)	0.0047 (11)
C17	0.0160 (11)	0.0170 (12)	0.0134 (11)	−0.0038 (9)	−0.0037 (8)	0.0042 (9)
C22	0.0167 (12)	0.0210 (12)	0.0142 (12)	−0.0033 (10)	−0.0007 (9)	0.0016 (10)
C9	0.0150 (11)	0.0161 (11)	0.0123 (11)	−0.0015 (9)	−0.0024 (9)	−0.0009 (9)
C18	0.0216 (12)	0.0175 (12)	0.0201 (12)	−0.0037 (10)	0.0009 (10)	−0.0028 (10)
C20	0.0170 (12)	0.0184 (13)	0.0238 (13)	−0.0057 (10)	−0.0071 (9)	0.0045 (10)
N11	0.0158 (10)	0.0201 (11)	0.0122 (9)	−0.0060 (8)	−0.0047 (7)	0.0018 (8)
C19	0.0168 (12)	0.0226 (13)	0.0227 (13)	−0.0041 (10)	0.0031 (10)	0.0007 (11)
C12	0.0207 (12)	0.0138 (12)	0.0126 (11)	0.0019 (9)	−0.0009 (9)	0.0000 (9)
C6	0.0177 (12)	0.0238 (13)	0.0161 (12)	−0.0032 (11)	−0.0076 (9)	0.0008 (10)
C3	0.0142 (11)	0.0145 (11)	0.0144 (11)	−0.0012 (9)	−0.0009 (9)	−0.0018 (10)
C4	0.0166 (11)	0.0168 (12)	0.0163 (11)	−0.0052 (9)	−0.0028 (9)	0.0006 (10)
C5	0.0229 (12)	0.0190 (12)	0.0147 (11)	0.0001 (10)	−0.0013 (9)	0.0036 (10)
C15	0.0164 (12)	0.0266 (13)	0.0179 (12)	−0.0010 (11)	−0.0026 (9)	−0.0030 (11)
C7	0.0173 (12)	0.0222 (13)	0.0188 (12)	−0.0079 (10)	−0.0029 (9)	0.0018 (10)

### Dichlorido{(E)-N,N-dimethyl-2-[phenyl(pyridin-2-yl)methylidene]hydrazine-1-carbothioamide}cadmium(II) Geometric parameters (Å, º)

**Table:**

Cd1—Cl2	2.4430 (6)	C17—C18	1.394 (3)
Cd1—S16	2.6001 (6)	C22—H22	0.9500
Cd1—Cl23	2.4832 (6)	C9—C3	1.484 (3)
Cd1—N8	2.352 (2)	C18—H18	0.9500
Cd1—N10	2.403 (2)	C18—C19	1.383 (4)
S16—C12	1.700 (3)	C20—H20	0.9500
N8—C3	1.349 (3)	C20—C19	1.384 (4)
N8—C7	1.331 (3)	N11—H11	0.8800
C21—H21	0.9500	N11—C12	1.363 (3)
C21—C22	1.387 (4)	C19—H19	0.9500
C21—C20	1.398 (4)	C6—H6	0.9500
N13—C14	1.462 (3)	C6—C5	1.384 (4)
N13—C12	1.332 (3)	C6—C7	1.389 (4)
N13—C15	1.467 (3)	C3—C4	1.384 (4)
N10—C9	1.285 (3)	C4—H4	0.9500
N10—N11	1.370 (3)	C4—C5	1.390 (3)
C14—H14A	0.9800	C5—H5	0.9500
C14—H14B	0.9800	C15—H15A	0.9800
C14—H14C	0.9800	C15—H15B	0.9800
C17—C22	1.400 (4)	C15—H15C	0.9800
C17—C9	1.485 (3)	C7—H7	0.9500
			
Cl2—Cd1—S16	104.61 (2)	C3—C9—C17	120.6 (2)
Cl2—Cd1—Cl23	109.64 (2)	C17—C18—H18	119.8
Cl23—Cd1—S16	108.43 (2)	C19—C18—C17	120.5 (2)
N8—Cd1—Cl2	99.39 (5)	C19—C18—H18	119.8
N8—Cd1—S16	140.28 (5)	C21—C20—H20	120.0
N8—Cd1—Cl23	92.25 (5)	C19—C20—C21	120.1 (2)
N8—Cd1—N10	67.36 (7)	C19—C20—H20	120.0
N10—Cd1—Cl2	120.90 (5)	N10—N11—H11	119.7
N10—Cd1—S16	73.20 (5)	C12—N11—N10	120.6 (2)
N10—Cd1—Cl23	127.43 (5)	C12—N11—H11	119.7
C12—S16—Cd1	102.86 (9)	C18—C19—C20	120.1 (2)
C3—N8—Cd1	119.79 (16)	C18—C19—H19	119.9
C7—N8—Cd1	121.53 (17)	C20—C19—H19	119.9
C7—N8—C3	118.5 (2)	N13—C12—S16	122.02 (19)
C22—C21—H21	120.1	N13—C12—N11	115.4 (2)
C22—C21—C20	119.9 (2)	N11—C12—S16	122.56 (19)
C20—C21—H21	120.1	C5—C6—H6	120.9
C14—N13—C15	115.5 (2)	C5—C6—C7	118.2 (2)
C12—N13—C14	121.7 (2)	C7—C6—H6	120.9
C12—N13—C15	122.7 (2)	N8—C3—C9	116.1 (2)
C9—N10—Cd1	120.77 (16)	N8—C3—C4	121.8 (2)
C9—N10—N11	118.6 (2)	C4—C3—C9	122.1 (2)
N11—N10—Cd1	120.60 (15)	C3—C4—H4	120.4
N13—C14—H14A	109.5	C3—C4—C5	119.2 (2)
N13—C14—H14B	109.5	C5—C4—H4	120.4
N13—C14—H14C	109.5	C6—C5—C4	119.0 (2)
H14A—C14—H14B	109.5	C6—C5—H5	120.5
H14A—C14—H14C	109.5	C4—C5—H5	120.5
H14B—C14—H14C	109.5	N13—C15—H15A	109.5
C22—C17—C9	118.4 (2)	N13—C15—H15B	109.5
C18—C17—C22	119.4 (2)	N13—C15—H15C	109.5
C18—C17—C9	122.2 (2)	H15A—C15—H15B	109.5
C21—C22—C17	120.0 (2)	H15A—C15—H15C	109.5
C21—C22—H22	120.0	H15B—C15—H15C	109.5
C17—C22—H22	120.0	N8—C7—C6	123.2 (2)
N10—C9—C17	123.7 (2)	N8—C7—H7	118.4
N10—C9—C3	115.7 (2)	C6—C7—H7	118.4
			
Cd1—S16—C12—N13	179.67 (19)	C22—C17—C9—C3	109.3 (3)
Cd1—S16—C12—N11	1.9 (2)	C22—C17—C18—C19	−0.3 (4)
Cd1—N8—C3—C9	−6.8 (3)	C9—N10—N11—C12	175.3 (2)
Cd1—N8—C3—C4	173.68 (18)	C9—C17—C22—C21	−179.3 (2)
Cd1—N8—C7—C6	−173.3 (2)	C9—C17—C18—C19	−179.7 (2)
Cd1—N10—C9—C17	173.71 (18)	C9—C3—C4—C5	−179.6 (2)
Cd1—N10—C9—C3	−3.0 (3)	C18—C17—C22—C21	1.2 (4)
Cd1—N10—N11—C12	−3.8 (3)	C18—C17—C9—N10	112.2 (3)
N8—C3—C4—C5	−0.1 (4)	C18—C17—C9—C3	−71.2 (3)
C21—C20—C19—C18	0.2 (4)	C20—C21—C22—C17	−1.5 (4)
N10—C9—C3—N8	6.4 (3)	N11—N10—C9—C17	−5.3 (4)
N10—C9—C3—C4	−174.1 (2)	N11—N10—C9—C3	178.0 (2)
N10—N11—C12—S16	0.9 (3)	C3—N8—C7—C6	1.8 (4)
N10—N11—C12—N13	−177.0 (2)	C3—C4—C5—C6	1.4 (4)
C14—N13—C12—S16	10.0 (3)	C5—C6—C7—N8	−0.5 (4)
C14—N13—C12—N11	−172.1 (2)	C15—N13—C12—S16	−167.47 (19)
C17—C9—C3—N8	−170.5 (2)	C15—N13—C12—N11	10.4 (3)
C17—C9—C3—C4	9.0 (4)	C7—N8—C3—C9	178.0 (2)
C17—C18—C19—C20	−0.4 (4)	C7—N8—C3—C4	−1.5 (4)
C22—C21—C20—C19	0.7 (4)	C7—C6—C5—C4	−1.2 (4)
C22—C17—C9—N10	−67.2 (3)		
